# Primary mucinous adenocarcinoma of the bladder with signet-ring cells: case report

**DOI:** 10.1590/S1516-31802007000500011

**Published:** 2007-09-02

**Authors:** Marcelo Lorenzi Marques, Gabriel Salum D'Alessandro, Daher Cezar Chade, Valéria Pereira Lanzoni, Cláudio José Ramos de Almeida

**Keywords:** Urinary bladder, Urinary bladder neoplasms, Mucinous adenocarcinoma, Immunohistochemistry, Signet ring cell carcinoma, Bexiga urinária, Neoplasias da bexiga urinária, Adenocarcinoma mucinoso, Imunohistoquímica, Carcinoma de células em anel de sinete

## Abstract

**CONTEXT:**

Primary adenocarcinomas of the bladder are uncommon and usually occur by contiguity with or hematogenic dissemination of other adenocarcinomas such as colorectal, prostate and gynecological tract carcinomas. Mucinous and signet-ring cell histological patterns are even rarer and it is often difficult to morphologically distinguish them from metastatic colorectal adenocarcinoma.

**CASE REPORT:**

We present and discuss a rare case of primary mucinous adenocarcinoma of the bladder with signet-ring cells in a 57-year-old male patient. Other primary sites for the tumor had been excluded and, in the absence of digestive tract tumor and for confirmation that it was a primary bladder tumor, an immunohistochemistry study was performed.

## RESUMO

**CONTEXTO:**

Adenocarcinomas vesicais primários são incomuns, o habitual é o comprometimento por contigüidade ou via hematogênica de outros adenocarcinomas como colorretal, próstata e trato ginecológico. O padrão histológico correspondente ao mucinoso e com células em anel de sinete é mais raro e, muitas vezes, há dificuldade em distingui-lo morfologicamente do adenocarcinoma colorretal metastático.

**RELATO DE CASO:**

Apresentamos e discutimos um caso de adenocarcinama mucinoso com células em anel de sinete primário da bexiga em um paciente masculino, de 57 anos. Foram excluídos outros sítios primários do tumor e, na ausência de tumor do trato digestivo e para confirmação de tumor vesical primário realizou-se estudo imunoistoquímico.

## INTRODUCTION

Bladder adenocarcinomas account for 0.5 to 2% of all malignant tumors of the bladder. The mucinous and signet-ring cell subtypes are rare and correspond to 20% of these tumors.^1,2^

Intramural tumor growth causes late symptoms. This delays the diagnosis and results in a poorer prognosis than for urothelial tumors.^1–3^

There are a variety of histological types among adenocarcinomas, including papillary, glandular, adenoid cystic, clear cell, mucinous and signet-ring cell carcinomas. The last of these types is characterized by the presence of intracellular vacuoles filled with mucin that displace the hyperchromatic nuclei.^2^

Because of the rarity of primary mucinous adenocarcinoma and the difficulties and particularities in diagnosing the primary tumor site, we present here a case of primary mucinous adenocarcinoma of the bladder with signet-ring cells.

## CASE REPORT

A 57-year-old male patient presented with a six-month history of painless intermittent macroscopic hematuria without coagula. The patient was a smoker and an alcoholic (Child B alcoholic liver cirrhosis). He presented weight loss, ascites and lower limb edema. There was no palpable abdominal tumor and digital rectal examination was normal.

**Figure 1:**
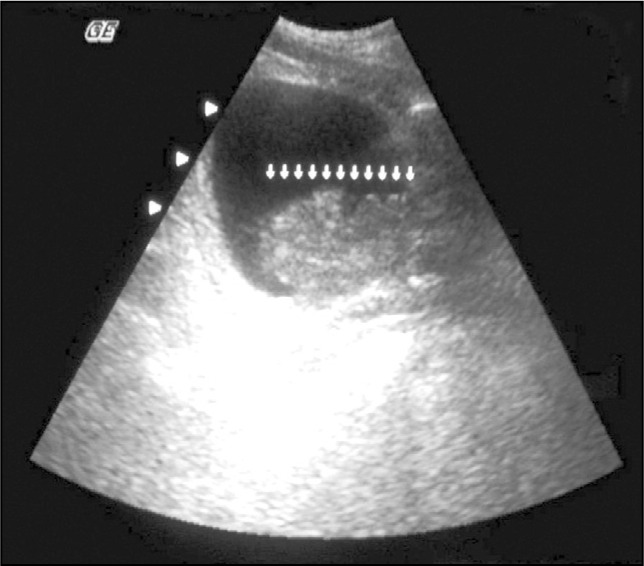
Abdominal ultrasound showing intravesical mass (10 cm in diameter).

**Figure 2:**
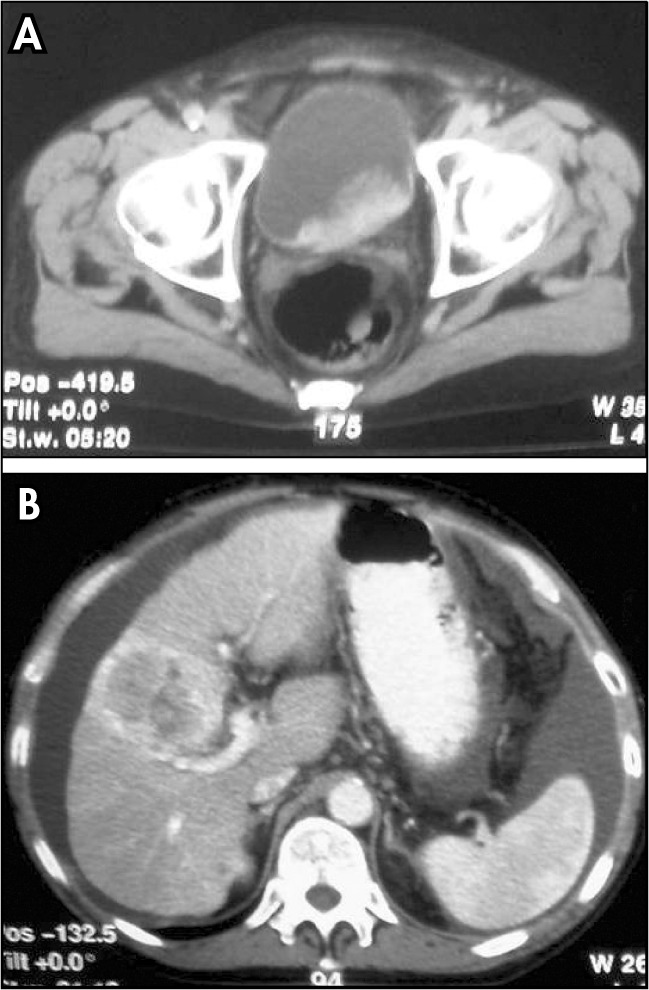
A) Computer tomography showing intravesical mass. B) Nodule measuring 7.5 cm in diameter in a cirrhotic liver suggesting metastasis.

Ultrasound and abdominal tomography revealed ascites, cirrhotic liver with nodules (largest nodule measuring 7.5 cm in diameter) and an intravesical mass of 10 cm in diameter that was continuous to the prostate and involved the floor and left lateral wall (Figures 1 and 2).

Cystoscopy made it possible to see a sessile mass in the left hemitrigone, involving the meatus, left lateral wall, posterior wall and part of the anterior wall, showing rough papillomatous areas and other areas with intact mucosa. Total resection of the intravesical tumor could not be performed because of its size and infiltration. On the other hand, the main objective of this procedure was diagnosis.

The histological findings were that this was a case of mucinous adenocarcinoma with signet-ring cells infiltrating the *lamina propria* and musculature, with preservation of the mucosa.

Because of the rarity of primary bladder tumors with these histological features, rigid rectosigmoidoscopy, colonoscopy, upper digestive endoscopy, pelvis resonance and laparoscopy together with liver biopsy were sequentially performed with the aim of detecting possible primary gastrointestinal cancer. Histopathological analysis of the liver fragment revealed mucinous adenocarcinoma associated with cirrhosis.

**Figure 3:**
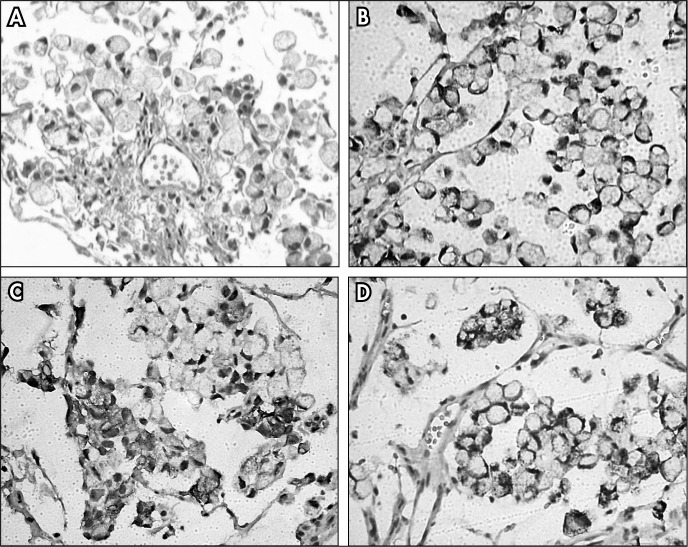
A) Mucinous adenocarcinoma of the bladder with signet-ring cells (hematoxylin-eosin, 200 x). B) Immunohistochemistry positive for Muc-2. C) Immunohistochemistry positive for CK7. D) Immunohistochemistry positive for CK20. (100 x).

In view of the absence of a digestive tract tumor and to confirm the primary bladder origin of the tumor, immunohistochemical analysis was performed. The tumor was found to be diffusely immunopositive for Muc-2 and p63 and focally immunopositive for CK7 and CK20, but negative for TTF-1, PSA, Muc-5AC and CDX-2. The immunohistochemical panel, together with the histological characteristics, characterized a mucus-secreting urothelial carcinoma (Figure 3).

## DISCUSSION

Primary bladder adenocarcinomas are uncommon and usually occur by contiguity with or hematogenic dissemination of other adenocarcinomas such as colorectal, prostate and gynecological tract carcinomas.^3^ The mucinous and signet-ring cell histological patterns are even rarer and it is often difficult to morphologically distinguish them from metastatic colorectal adenocarcinoma.

Immunohistochemical analysis using a panel of specific markers is an important alternative for etiological differentiation of these tumors. A CDX-2 and CK20-positive and CK7-negative profile is indicative of digestive tract adenocarcinoma, particularly colorectal carcinoma, and is rare in urothelial tumors, which normally express CK7 alone or together with CK20^3^ and do not express CDX-2.

Few studies on the expression of the cytokeratins CK20 and CK7 in primary bladder adenocarcinoma cases are available in the literature. Torenbeek et al.^4^ observed the expression, at least focally, of CK7 in 82% of cases and CK20 in 73%, whereas a CK20-positive and CK7-negative profile was detected in only 29% of the cases of primary adenocarcinomas of the bladder.^3^

The expression of markers for mucin-producing tumors such as Muc-2 and Muc-5AC is very heterogeneous and is observed in a wide variety of tumors, particularly those originating from the intestinal tract.^5^ Immunohistochemical evaluation of these specific markers to help in diagnosing carcinomas for which the primary origin is uncertain is not usually recommended.

## CONCLUSIONS

Primary adenocarcinoma of the bladder is very rare and its precise diagnosis through conventional methods is difficult to achieve. For this reason, immunohistochemical analysis has major importance in determining the final diagnosis. In this case, the panel was immunopositive for Muc-2, CK7 and CK20 and immunonegative for CDX-2; this, together with the histological findings, characterized a mucus-secreting urothelial carcinoma. However, the markers for mucin-producing tumors, such as Muc-2 and Muc-5AC, are also observed in a wide variety of other tumors. Therefore, investigation of these markers is not usually recommended.
